# Sex differences in mandibular repositioning device therapy effectiveness in patients with obstructive sleep apnea syndrome

**DOI:** 10.1007/s11325-018-1766-8

**Published:** 2018-12-22

**Authors:** Marie-Françoise Vecchierini, Valérie Attali, Jean-Marc Collet, Marie-Pia d’Ortho, Frederic Goutorbe, Jean-Baptiste Kerbrat, Damien Leger, Florent Lavergne, Christelle Monaca, Pierre-Jean Monteyrol, Laurent Morin, Eric Mullens, Bernard Pigearias, Francis Martin, Hauria Khemliche, Lionel Lerousseau, Jean-Claude Meurice, on behalf of the ORCADES investigators, Darius Abedipour, Aurélie Allard-Redon, Alexandre Aranda, Valérie Attali, Frédérique Bavozet, Martine Becu, Wally Beruben, Jerome Bessard, Isabelle Bonafe, Mohammed Boukhana, Bruno Chabrol, Gérard Chatte, Chauvel Lebret, Jean-Marc Collet, Olivier Coste, Nathalie Dumont, Sophie Durand-Amat, Marie-Pia D’ortho, Jean Marc Elbaum, Olivier Gallet De Santerre, Frédéric Goutorbes, Thierry Grandjean, Wilma Guyot, Doniphan Hammer, Carmen Havasi, Pascal Huet, Jean Baptiste Kerbrat, Hauria Khemliche, Christian Koltes, Damien Leger, Laurent Lacassagne, Xavier Laur, Lionel Lerousseau, Olivier Liard, Christophe Loisel, Matthieu Longuet, Anne Mallart, Francis Martin, Frédéric Merle Beral, Jean Claude Meurice, Zoubida Mokhtari, Christelle Monaca, Pierre Jean Monteyrol, Jean-François Muir, Eric Mullens, Dominique Muller, Charles Paoli, François Xavier Petit, Bernard Pigearias, Marc Pradines, Arnauld Prigent, Gil Putterman, Marc Rey, Mickael Samama, Renaud Tamisier, Michel Tiberge, Cyrille Tison, Fabienne Tordjman, Bernard Triolet, Christian Vacher, Marie-Françoise Vecchierini, Alain Verain

**Affiliations:** 1 AP-HP, Centre du Sommeil et de la Vigilance, Hôpital Hôtel Dieu, Paris, France; 2 Sorbonne Paris Cité, Université Paris Descartes, Paris, France; 3 Service des Pathologies du Sommeil (Département “R3S”), AP-HP groupe hospitalier Pitié-Salpêtrière Charles Foix, Paris, France; 4 INSERM, UMRS1158 Neurophysiologie Respiratoire Expérimentale et Clinique, Sorbonne Université, Paris, France; 5 AP-HP, Stomatologie et Chirurgie Maxillo-Faciale, Groupe Hospitalier Pitié-Salpêtrière Charles Foix, Paris, France; 6 AP-HP, DHU FIRE, Physiologie et Explorations Fonctionnelles, Hôpital Bichat-Claude Bernard, Paris, France; 7 UFR de Médecine, Université Denis Diderot Paris 7, Paris, France; 8 Centre Médecine du Sommeil, Centre Hospitalier de Béziers, Béziers, France; 9 Stomatologie et Chirurgie Maxillo-Faciale, Hôpital Charles Nicolle, Rouen, France; 10 ResMed Science Center, 292, Allée Jacques Monod, 69791 Saint-Priest CEDEX, France; 11 Neurophysiologie Clinique, Hôpital Roger Salengro, Lille, France; 12 Polyclinique du Tondu, Oto-Rhino-Laryngologie, Bordeaux, France; 13 Fondation Bon Sauveur, Laboratoire du Sommeil, Albi, France; 14 Laboratoire du Sommeil, Nice, France; 15 Groupe Hospitalier Public Sud de l’Oise, Senlis, France; 16 Service de Pneumologie, Centre Hospitalier Antibes, Antibes, France; 17 Pneumologie, Centre Hospitalier Universitaire, Poitiers, France

**Keywords:** Obstructive sleep apnea, Mandibular repositioning device, Sex differences, Apnea-hypopnea index

## Abstract

**Purpose:**

Mandibular repositioning devices (MRDs) are an effective treatment option for obstructive sleep apnea syndrome (OSAS), particularly in patients who refuse or cannot tolerate continuous positive airway pressure (CPAP). However, sex differences in the response to therapy and predictors of response are not clearly defined. This analysis of data from the long-term prospective ORCADES trial compared MRD efficacy in men and women with OSAS.

**Methods:**

The ORCADES study included patients with newly diagnosed mild-to-moderate or severe OSAS who refused or were non-compliant with CPAP. MRD therapy was titrated over 3–6 months. The primary endpoint was treatment success (≥ 50% decrease in apnea-hypopnea index (AHI)). Complete response was defined using a range of AHI cut-off values (< 5/h, < 10/h, < 15/h).

**Results:**

Overall treatment success rates were 89% in women and 76% in men (*p* = 0.019); corresponding rates in those with severe OSAS (AHI > 30/h) were 100% and 68% (*p* = 0.0015). In women vs. men, overall complete response rates at AHI cut-off values of < 5/h, <10/h, and < 15/h were 49 vs. 34% (*p* = 0.0052), 78 vs. 62% (*p* = 0.016), and 92 vs. 76% (*p* = 0.0032). On multivariate analysis, significant predictors of MRD treatment success were overbite and baseline apnea index in men, and neck circumference and no previous CPAP therapy in women. There were sex differences in the occurrence of side effects. Temporomandibular joint pain was the most common reason for stopping MRD therapy.

**Conclusions:**

MRD therapy was effective in women with OSA of any severity, with significantly higher response rates compared with men especially in severe OSAS.

**Trial registration:**

www.clinicaltrials.gov (NCT01326143).

## Introduction

Obstructive sleep apnea syndrome (OSAS) is characterized by repetitive complete or partial occlusions of the upper airway with persistent inspiratory efforts during sleep, followed by oxyhemoglobin desaturations and terminated by arousals. OSAS is a public health burden because of its medical and socioeconomic consequences, including a higher likelihood of vehicle crashes and occupational accidents, increased risk of cardiovascular diseases, neurocognitive dysfunction, and impaired quality of life [1].

The gold standard treatment for OSAS is continuous positive airway pressure (CPAP), which has been shown to reduce sleepiness [2] and road accidents [3], and might decrease cardiovascular risk and mortality [4]. However, compliance with CPAP is an issue in up to half of all users [5], potentially limiting its effectiveness [6]. Mandibular repositioning devices (MRDs) enlarge the upper airway during sleep by holding the mandible in a forward position and are an effective alternative to CPAP, particularly in mild-to-moderate OSA or in patients not adherent to or refusing CPAP [7]. Reductions in the apnea-hypopnea index (AHI) during MRD therapy are usually smaller than those during CPAP, but patient acceptability and compliance may be better, with similar quality of life and symptom benefits [8].

The reported prevalence of OSAS is generally lower in women vs. men, and there are a number of sex-related differences in disease manifestation and presentation [1]. Mechanisms underlying sex differences in OSAS prevalence are not fully understood [9, 10], and there is a relative lack of data on sex differences in the response to OSA therapies, particularly MRDs.

The prospective ORthèse d’avanCée mAndibulaire type O.R.M dans le traitement en DEuxième intention du SAHOS sévère (ORCADES) cohort study is investigating the long-term efficacy and tolerability of a computer-aided design (CAD)/computer-aided manufacturing (CAM) MRD in OSA patients non-compliant with or intolerant of CPAP. Interim short-term results showed that MRD treatment was effective across all severities of OSAS, and univariate logistic regression analysis of factors predicting efficacy indicated better MRD efficacy in women than in men (hazard ratio 2.12, 95% confidence interval 1.21–3.73; *p* = 0.0078), although this did not persist in multivariate analysis [11]. This post hoc analysis of the ORCADES study compared CAD/CAM MRD efficacy in men and women after 3–6 months follow-up.

## Methods

### Study design and oversight

The prospective, observational ORCADES study (NCT01326143) was conducted at 28 centers in France. The study design and details of MRD devices have been described in detail previously [11]. The Steering Committee (SC) defined the study design and was responsible for the clinical and scientific conduct of the study and publication of the results. C.R.O. Clinact (France) performed the data collection, quality control, management, and analysis. The SC had full access to all data and takes responsibility for the integrity and accuracy of the analysis.

### Patients

Eligible patients had newly diagnosed mild-to-moderate (AHI 5–30/h) or severe (AHI > 30/h) OSAS, excessive daytime sleepiness (Epworth Sleepiness Scale (ESS) score > 10), and refusal of or non-compliance with CPAP. Exclusion criteria included previous MRD treatment, contraindications to MRD therapy, central apnea index > 5/h, severe sleep comorbidities other than OSAS, and coexisting psychiatric disease.

### MRD titration and follow-up

Patients were fitted with a CAD/CAM MRD (Narval CC™; ResMed). Mandibular advancement was gradually adjusted at the discretion of the dental sleep specialist (over a 15-mm range) until the best benefit-risk ratio between symptom resolution and tolerability was achieved. At titration visits, patients reported the degree of improvement (none, some, important) in three symptoms (snoring, fatigue, and sleepiness) and also rated tolerability (based on articular, dental, and periodontal pain) on a non-graduated, 10-cm visual analogue scale.

### Assessments and endpoints

The primary endpoint was treatment success (proportion of patients with a ≥ 50% decrease in AHI from baseline to follow-up). Complete response was defined using a range of AHI cut-off values (< 5/h, < 10/h, < 15/h).

At baseline and follow-up visits, self-reported clinical symptoms were assessed, sleep quality, subjective sleepiness was scored using the ESS, quality of life using the Quebec Sleep Questionnaire (QSQ) and fatigue using the Pichot scale [11].

Sleep and/or nocturnal respiratory parameters were recorded at baseline and after 3 months with the same polygraphy (PG) or polysomnography (PSG) device used to diagnose OSA. If AHI decreased by < 50% and/or symptoms persisted, PSG/PG was performed again at 6 months after additional mandibular advancements. PSG/PG recordings were manually scored using American Academy of Sleep Medicine guidelines [12]. Positional OSA was defined when the supine AHI was at least twice that in other positions and AHI was > 10/h [13].

Self-reported MRD compliance (hours/night; nights/week) was reported at each follow-up visit, and comprehensive data on MRD-related side effects were collected*.* Side effect severity and impact on MRD treatment was determined by sleep and dental sleep physicians.

### Statistical analysis

Quantitative changes from baseline to follow-up were presented as mean ± standard deviation and compared using unpaired or paired Student’s *t* test or nonparametric test according to normality of distribution and group comparison. Qualitative changes were described using frequency distribution and compared using Fisher Exact or chi-square test. Comparisons between men and women were assessed using Student’s *t* test, ANOVA, or Wilcoxon-Mann-Whitney test. Three logistic models were created and backward stepwise regression analysis was used to determine independent factors associated with therapy success and complete response, model 1: all patients; model 2: men; and model 3: women. For all models, variables with a *p* value < 0.10 in the univariate analysis were entered in the stepwise logistic regressions, and variables with a *p* value < 0.05 were retained in the final models. For model 1, univariate analysis on the interaction between gender and potential predictive factors was also performed. Statistical analyses were performed using SAS version 9.

## Results

### Study population

**Fig. 1:**
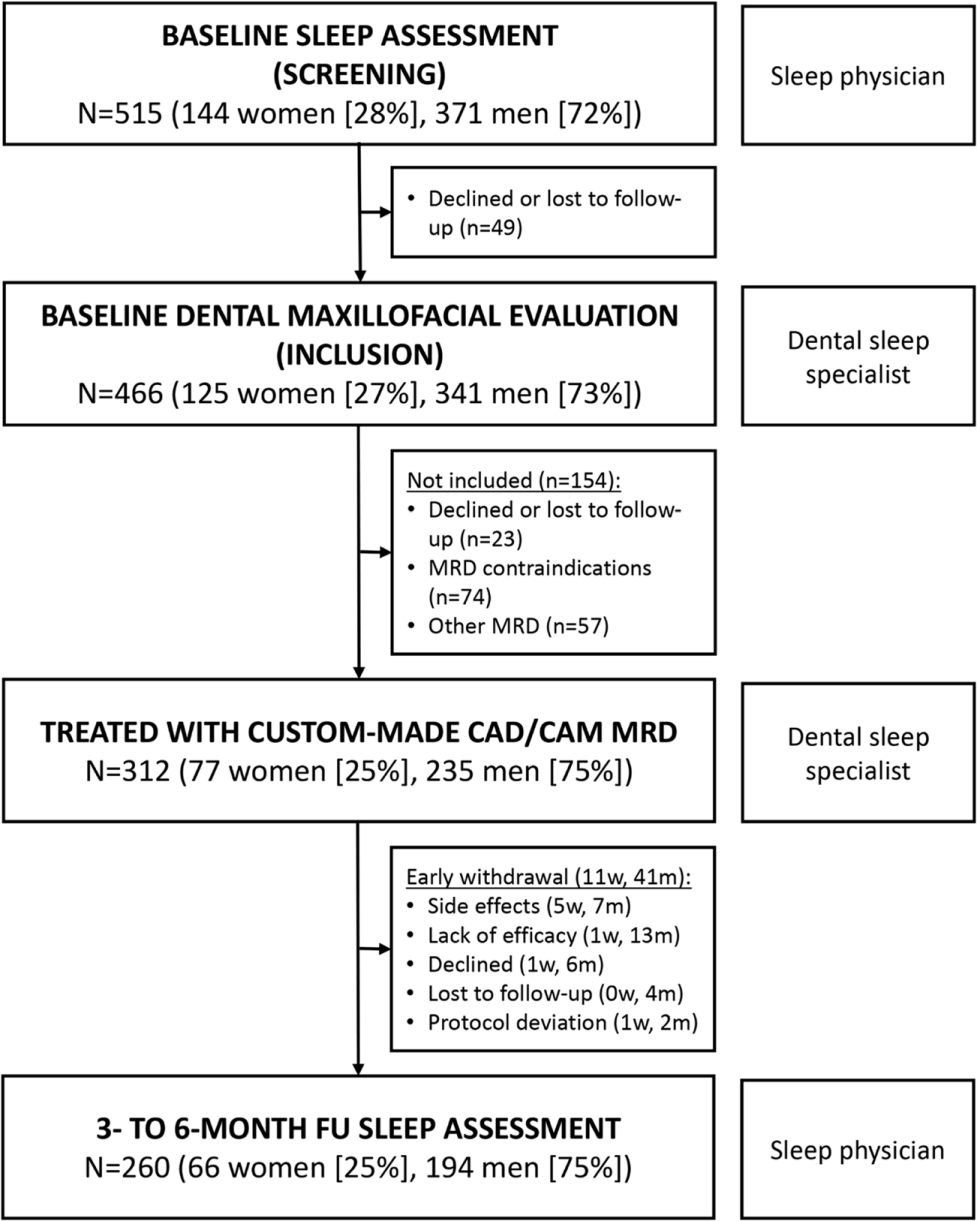
ORCADES study flow chart. *CAD/CAM* computer-aided design/computer-aided manufacturing, *FU* follow-up, *m* men, *MRD* mandibular repositioning device, *w* women

A total of 515 eligible OSA patients (144 women, 371 men) were screened between May 2011 and September 2013; 154 with contraindications to MRD therapy or treatment with another MRD or who declined to participate were excluded. Therefore, 312 eligible patients (77 women, 235 men) were enrolled in our cohort study. Of these, 52 patients withdrew from the study before evaluation of the endpoint criteria, leaving 260 available for follow-up analysis (Fig. 1). There were significantly fewer women than men in the study population and there were a number of statistically significant between-group differences (Table 1). Women were older; had a lower body mass index (BMI), neck and waist circumference, and diastolic blood pressure; were more likely to have retrognathia, hypothyroidism, and positional OSA; and had a lower apnea index (AI) and non-supine AHI vs. men (Table 1). Women and men reported similar MRD use (6.7 and 6.6 h/night on 6.8 and 6.5 nights/week, respectively). Device use every night was reported by 85% of women and men, and device use ≥ 4 h/night on ≥ 4 days/week by 100% of women and 94% of men. Women and men required a similar number of titration visits to optimize MRD efficacy (1.8 ± 1.2), with similar final mean mandibular advancement (6.8 ± 2.2 vs. 7.4 ± 2.1 mm; *p* = 0.07) and percentage of maximal mandibular advancement (median (Q1, Q3) 76.4% (66.7, 100) vs. 83.3% (66.7, 100); *p* = 0.26).

**Table 1:** Baseline characteristics by patient sex

*N* = 312	Women	Men	*p* value
Number (%)	77 (24.7)	235 (75.3)	< 0.0001
Age, years	56.8 ± 10.2	52.1 ± 11.2	0.0013
Body mass index, kg/m^2^	26.7 ± 5.4	27.2 ± 3.5	0.032
Obese, *N* (%)	17 (22.1)	44 (18.7)	NS
Waist circumference, cm	90.5 ± 13.4	98.9 ± 11.2	< 0.0001
Neck circumference, cm	35.6 ± 3.1	41.0 ± 3.1	< 0.0001
Retrognathia, *N* (%)	33 (42.9)	47 (20)	0.0006
Maximum mandibular advancement, mm	8.7 ± 2.5	8.9 ± 2.4	NS
No previous CPAP therapy, *N* (%)	45 (58.4)	107 (45.7)	NS
Systolic blood pressure, mmHg	124.7 ± 13.1	127.6 ± 11.2	NS
Diastolic blood pressure, mmHg	74.6 ± 10.2	78.9 ± 9.7	0.0032
Comorbidities, *N* (%)			
Arterial hypertension	25 (32.5)	69 (29.4)	NS
Diabetes	5 (6.5)	16 (6.8)	NS
Hypothyroidism	11 (14.3)	4 (1.7)	< 0.0001
Stroke	3 (3.9)	4 (1.7)	NS
Restless leg syndrome	0 (0)	4 (1.7)	NS
Respiratory parameters			
Mean AHI, /h	26.5 ± 13.7	30.1 ± 15.1	NS
Mild OSA (AHI 5–15/h), *N* (%)	14 (18.2)	35 (14.9)	NS
Moderate OSA (AHI 15–30/h), *N* (%)	37 (48.1)	94 (40.0)	NS
Severe OSA (AHI > 30/h), *N* (%)	26 (33.8)	106 (45.1)	0.08
Supine AHI, /h	32.2 ± 17.9	38.5 ± 23.2	NS
Non-supine AHI, /h	13.1 ± 15.3	19.6 ± 17.9	0.0038
Positional OSA, *N* (%)	45 (58)	82 (35)	0.0026
AI, /h	9.2 ± 9.0	13.8 ± 13.0	0.0044
HI, /h	17.2 ± 10.1	16.4 ± 9.7	NS
cAI, /h	0.3 ± 0.8	0.5 ± 1.0	NS
SpO_2_, %	93.8 ± 1.9	93.7 ± 1.9	NS
Minimum SpO_2_, %	83.0 ± 6.8	81.3 ± 8.0	NS
Time with SpO_2_ < 90%, min	21.0 ± 32.1	26.5 ± 55.7	NS
ODI, /h	21.8 ± 19.3	21.7 ± 18.1	NS
Snoring, /h	108 ± 146	143 ± 276	NS
Snoring duration, % TRT	27%	29%	NS

*AHI* apnea-hypopnea index, *AI* apnea index, *cAI* central apnea index, *CPAP* continuous positive airway pressure, *HI* hypopnea index, *NREM* non-rapid eye movement, *NS* not significant, *ODI* oxygen desaturation index, *OSA* obstructive sleep apnea, *REM* rapid eye movement, *SpO2* oxygen saturation

Values are mean ± standard deviation, or number of patients (%)

### Primary endpoint: MRD efficacy

**Fig. 2:**
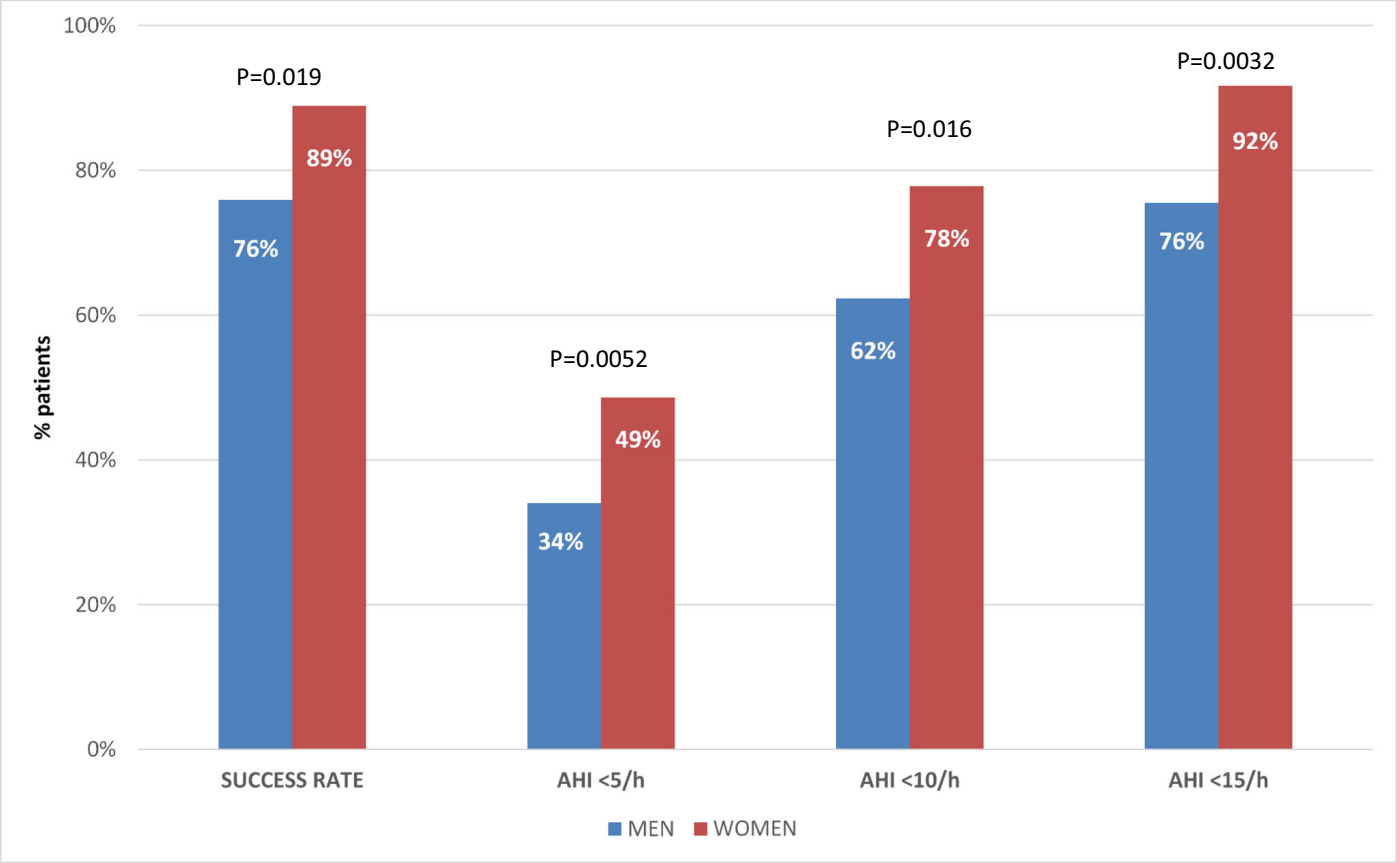
Mandibular repositioning device efficacy in men and women at 3- to 6-month follow-up. *AHI* apnea-hypopnea index, *Success rate* percentage of patients with a ≥ 50% decrease in AHI from baseline to follow-up

Overall treatment success and complete response rates were significantly higher in women than in men (Fig. 2), primarily due to significant sex differences in the subgroup with severe OSA (success rate 100% in women vs. 67.7% in men, *p* = 0.0015; complete response at AHI < 5/h, < 10/h, and < 15/h in 49 vs. 34.0% (*p* = 0.0052), 78 vs. 62%(*p* = 0.016), and 92 vs. 76% (*p* = 0.0032), respectively).

### Secondary endpoints: sleep and respiratory parameters

There was a significant reduction in mean AHI, irrespective of sex (Table 2), again due to a significantly greater reduction in the severe OSA subgroup (Table 3). Mean AI, hypopnea index (HI), supine and non-supine AHI, nadir oxygen saturation (SpO_2_), and time with SpO_2_ < 90% decreased significantly from baseline to follow-up in men and women (Table 2), with the greatest decrease in HI seen in women with severe OSA (Table 3).

**Table 2:** Changes in respiratory parameters during MRD therapy by patient sex

	Baseline	Follow-up	Difference	*p* value	
				vs. Baseline	Men vs. women
AHI, /h					
Women	26.5 ± 13.7	6.5 ± 5.4	− 19.3 ± 11.7	< 0.0001	NS
Men	30.1 ± 15.1	11.6 ± 13.2	− 18.7 ± 12.6	< 0.0001	
AI, /h					
Women	9.2 ± 9.0	1.4 ± 2.3	− 7.5 ± 8.4	< 0.0001	0.084
Men	13.8 ± 13.0	4.5 ± 9.2	− 9.7 ± 10.7	< 0.0001	
HI, /h					
Women	17.2 ± 10.1	5.1 ± 4.4	− 11.4 ± 8.7	< 0.0001	NS
Men	16.4 ± 9.74	7.2 ± 7.73	− 9.0 ± 10.4	< 0.0001	
Supine AHI, /h					
Women	32.2 ± 17.9	7.6 ± 6.8	− 22.7 ± 15.7	< 0.0001	NS
Men	38.5 ± 23.2	13.8 ± 17.81	− 24.3 ± 23.0	<0.0001	
Non-supine AHI, /h					
Women	13.1 ± 15.3	4.0 ± 4.7	− 8.9 ± 15.7	0.0075	NS
Men	19.6 ± 17.9	7.1 ± 12.2	− 12.5 ± 14.9	< 0.0001	
Mean SpO_2_, %					
Women	93.8 ± 1.9	94.0 ± 1.9	0.1 ± 1.5	NS	NS
Men	93.7 ± 1.9	93.9 ± 1.92	0.2 ± 1.8	NS	
Nadir SpO_2_, %					
Women	83.0 ± 6.8	85.3 ± 8.9	2.3 ± 9.0	< 0.0001	NS
Men	81.3 ± 8.0	84.6 ± 8.9	3.3 ± 9.9	< 0.0001	
SpO_2_ < 90%, min					
Women	21.0 ± 32.1	10.7 ± 22.8	− 7.3 ± 29.2	0.0018	NS
Men	26.5 ± 55.7	18.0 ± 52.7	− 9.1 ± 57.3	< 0.0001	
ODI, /h					
Women	21.8 ± 19.3	6.7 ± 6.1	− 14.1 ± 16.1	< 0.0001	NS
Men	21.7 ± 18.1	10.3 ± 12.9	− 11.7 ± 18.7	< 0.0001	
Time in supine position, min					
Women	230 ± 108	221 ± 132	− 51 ± 99	0.016	0.0053
Men	187 ± 108	205 ± 110	18 ± 118	0.07	
Time in non-supine position, min					
Women	194 ± 135	201 ± 123	35 ± 92	NS	0.027
Men	236 ± 115	217 ± 114	− 25 ± 124	0.07	

Values are mean ± standard deviation

*AHI* apnea-hypopnea index, *AI* apnea index, *HI* hypopnea index, *NS* not significant, *ODI* oxygen desaturation index, *SpO2* oxygen saturation

**Table 3:** Change in respiratory parameters during MRD therapy by patient sex and sleep apnea severity

	Number	Baseline	Number	Follow-up	Difference	*p* value	
						Vs. baseline	Men vs. women
AHI, /h							
Women, mild-to-moderate OSA	51	18.9 ± 7.3	48	6.5 ± 5.4	− 19.3 ± 11.7	< 0.0001	NS
Men, mild-to-moderate OSA	129	19.4 ± 6.3	114	11.6 ± 13.20	− 18.7 ± 12.6	< 0.0001	
Women, severe OSA	26	41.4 ± 10.5	22	8.7 ± 6.37	− 32.2 ± 8.2	< 0.0001	0.011
Men, severe OSA	106	43.2 ± 12.1	96	18.3 ± 16.25	− 25.0 ± 14.8	< 0.0001	
AI, /h							
Women, mild-to-moderate OSA	50	6.2 ± 6.1	47	1.3 ± 2.6	− 4.6 ± 5.8	< 0.0001	NS
Men, mild-to-moderate OSA	129	7.1 ± 5.8	114	1.6 ± 2.9	− 5.5 ± 5.2	< 0.0001	
Women, severe OSA	26	15.1 ± 10.7	22	1.8 ± 1.5	− 13.4 ± 10.0	< 0.0001	NS
Men, severe OSA	106	21.8 ± 14.6	96	7.9 ± 12.5	− 14.7 ± 13.2	< 0.0001	
HI, /h							
Women, mild-to-moderate OSA	50	12.9 ± 6.7	47	4.2 ± 3.3	− 8.7 ± 6.4	< 0.0001	NS
Men, mild-to-moderate OSA	129	12.3 ± 6.9	114	4.5 ± 4.2	− 7.9 ± 7.6	< 0.0001	
Women, severe OSA	26	25.4 ± 10.6	22	6.9 ± 5.7	− 17.2 ± 10.1	< 0.0001	0.026
Men, severe OSA	106	21.3 ± 10.4	96	10.4 ± 9.5	− 10.4 ± 12.9	< 0.0001	
Supine AHI, /h							
Women, mild-to-moderate OSA	37	24.7 ± 11.9	42	6.8 ± 5.5	− 18.0 ± 12.4	< 0.0001	NS
Men, mild-to-moderate OSA	98	29.7 ± 19.2	93	7.5 ± 7.8	− 21.7 ± 18.1	< 0.0001	
Women, severe OSA	19	46.9 ± 18.7	17	9.3 ± 9.3	− 33.8 ± 17.6	< 0.0001	NS
Men, severe OSA	79	49.5 ± 23.2	77	21.4 ± 22.9	− 27.2 ± 27.2	< 0.0001	
Non-supine AHI, /h							
Women, mild-to-moderate OSA	22	9.7 ± 10.8	22	3.3 ± 4.8	− 6.3 ± 10.0	0.0008	NS
Men, mild-to-moderate OSA	46	11.7 ± 12.2	51	3.4 ± 4.5	− 8.3 ± 13.2	< 0.0001	
Women, severe OSA	10	20.5 ± 21.0	9	5.6 ± 4.1	− 17.7 ± 25.6	NS	NS
Men, severe OSA	54	26.3 ± 19.3	43	11.5 ± 16.4	− 16.2 ± 15.5	< 0.0001	
Time in supine position, min							
Women, mild-to-moderate OSA	25	220 ± 96	21	232 ± 121	− 26 ± 87	NS	0.033
Men, mild-to-moderate OSA	53	159 ± 99	49	192 ± 108	26 ± 126	0.032	
Women, severe OSA	11	254 ± 133	10	199 ± 157	− 113 ± 105	0.016	0.008
Men, severe OSA	58	212 ± 111	46	219 ± 111	12 ± 112	NS	
Time in non-supine position, min							
Women, mild-to-moderate OSA	25	214 ± 143	21	213 ± 121	22 ± 86	NS	NS
Men, mild-to-moderate OSA	52	270 ± 95	49	229 ± 114	− 27 ± 130	NS	
Women, severe OSA	9	137 ± 92	9	173 ± 128	78 ± 105	NS	0.061
Men, severe OSA	57	205 ± 124	46	204 ± 114	− 23 ± 120	NS	

Values are mean ± standard deviation

*AHI* apnea-hypopnea index, *AI* apnea index, *HI* hypopnea index, *Mild-to-moderate* AHI 5–30/h, *NS* not statistically significant, *Severe* AHI > 30/h

The ESS score decreased significantly during MRD therapy (from 10.6 ± 5.3 to 7.5 ± 4.4 in women and 11.4 ± 4.7 to 7.9 ± 4.3 in men; *p* < 0.0001) with no sex differences. Excessive daytime sleepiness improved in 62% of men and women. Most clinical symptoms improved significantly in men and women after 3–6 months of MRD treatment; snoring disappeared in 53% of women and 48% of men. Self-reported symptoms, including sleep quality, awakening, nocturia, libido disorders, and mouth breathing, improved similarly in women and men. However, reduction of morning headache was more pronounced in women vs. men (*p* = 0.05). Mean total and domain scores on the QSQ significantly improved from baseline under MRD treatment (+ 24%, *p* < 0.0001), and were similar in men and women. The Pichot fatigue scale score also improved significantly and similarly in men and women (− 35%, *p* < 0.0001).

Among the 149 patients who underwent PSG, there were no significant changes in total sleep time, sleep latency, N1 + N2 sleep, slow wave sleep, rapid eye movement sleep duration, and wake duration during sleep from baseline to follow-up, and no differences between women and men. Arousals decreased to a similar extent in men and women (*p* < 0.0001 vs. baseline). Women slept less in the supine position during MRD therapy compared with baseline (Table 2); this was more marked in severe OSA (Table 3).

### Other parameters

Body weight, BMI, blood pressure, and neck/waist circumference did not change significantly during MRD use, apart from a significant increase in waist circumference in men (1.34 ± 5.63 cm, *p* = 0.0003 vs. baseline).

### Factors predictive of MRD efficacy

Model 1 (whole population) identified two significant independent predictors of MRD treatment success: initial AI and overbite, with no interactions by gender. A 10/h decrease in AI and a 1-mm increase in overbite were associated with a 41 and 43% increase in the number of MRD responders, respectively. No significant predictors of complete response in the overall patient population were identified. For models 2 (men) and 3 (women), univariate analysis identified a number of significant predictive factors (Table 4). Significant independent predictors of MRD treatment success are summarized in Table 5. In men, a 10/h decrease in AI and a 1-mm increase in overbite were associated with a 50 and 48% increase in the number of MRD responders, respectively. In women, treatment success probability was increased by 27% by a 1-mm increase in mandibular advancement. The similarity of independent predictors of treatment success overall and in men is probably due to the fact that men made up 75% of the total study population. For complete response, significant independent predictors included AHI in men, and neck circumference and no previous CPAP therapy in women (Table 5).

**Table 4:** Univariate analysis of factors predicting therapy success and complete response (AHI < 10/h) in men and women

Variable	OR (95% CI)	*p* value
Treatment success, men		
Neck circumference (cm)	0.88 (0.78; 0.99)	0.033
Waist circumference (cm)	0.95 (0.92; 0.98)	0.001
Obesity	0.30 (0.12; 0.75)	0.01
Dental class (class II vs. I)	3.45 (1.16; 10.25)	0.07
Dental class (class III vs. I)	1.63 (0.34; 7.88)	0.07
Overbite (mm)	1.51 (1.19; 1.89)	0.0005
Maximum mandibular advancement (mm)	1.18 (1.03; 1.37)	0.02
Overjet (mm)	1.32 (1.06; 1.65)	0.01
Initial AHI (/h)	0.97 (0.95; 0.98)	0.0013
Initial AI (/h)	0.95 (0.93; 0.98)	< 0.0001
Initial supine AHI (/h)	0.98 (0.97; 0.99)	0.02
Mouth breathing (yes/no)	2.14 (0.96; 4.74)	0.06
Complete response, men		
Neck circumference (cm)	0.81 (0.72; 0.91)	0.0003
Waist circumference (cm)	0.94 (0.92; 0.97)	0.0002
Neck circumference/waist circumference ratio	∞ (0.91;∞)	0.05
Obesity	0.24 (0.10; 0.58)	0.037
Dental class (class II vs. I)	2.45 (1.09; 5.51)	0.07
Dental class (class III vs. I)	1.84 (0.47; 7.22)	0.07
Overbite (mm)	1.39 (1.15; 1.67)	0.0007
Overjet (mm)	1.19 (0.99; 1.43)	0.06
Initial AHI (/h)	0.91 (0.89; 0.94)	< 0.0001
Initial AI (/h)	0.91 (0.89; 0.94)	< 0.0001
Initial HI (/h)	0.96 (0.93; 0.99)	0.006
Initial supine AHI (/h)	0.96 (0.94; 0.98)	< 0.0001
No previous treatment by CPAP (yes/no)	3.24 (1.77; 5.95)	0.0001
Mouth breathing (yes/no)	1.77 (0.91;3.44)	0.094
Treatment success, women		
Vertical dimension (mm)	1.18 (1.00; 1.39)	0.05
Maximum mandibular advancement (mm)	1.73 (1.08; 2.78)	0.02
Mandibular advancement (%)	0.97 (0.94; 0.99)	0.03
Initial AHI (/h)	1.10 (1.01; 1.21)	0.03
Initial HI (/h)	1.11 (0.99; 1.24)	0.07
Initial supine AHI (/h)	1.07 (0.99; 1.16)	0.08
Complete response, women		
Neck circumference (cm)	0.83 (0.68; 1.03)	0.089
Mandibular advancement (%)	0.98 (0.96; 1.0)	0.09
Initial AHI (/h)	0.94 (0.89; 0.99)	0.01
Initial AI (/h)	0.94 (0.88; 1.0)	0.05
Initial HI (/h)	1.11 (0.99; 1.24)	0.07
No previous CPAP treatment (yes/no)	7.64 (1.87; 31.33)	0.005

*AHI* apnea-hypopnea index, *AI* apnea index, *HI* hypopnea index

**Table 5:** Factors predicting short-term MRD efficacy on multivariate analysis

	Odds ratio	95% CI	*p* value
Whole population			
Success			
Overbite	1.43	1.09–1.87	0.0096
Baseline apnea index	0.96	0.94–0.98	0.025
Men			
Success			
Overbite	1.48	1.16–1.88	0.0015
Baseline apnea index	0.95	0.93–0.98	0.0002
Complete response			
Baseline AHI	0.91	0.88–0.93	< 0.0001
Women			
Success			
Maximum mandibular advancement	1.72	1.07–2.77	0.0236
Complete response			
Neck circumference	0.75	0.58–0.96	0.0265
No previous CPAP therapy	11.43	2.28–57.33	0.0031

*AHI* apnea-hypopnea index, *CI* confidence interval, *Complete response* AHI < 10/h, *CPAP* continuous positive airway pressure, *Success* ≥ 50% decrease in the AHI

### Tolerability

At least one side effect was reported by 55% of women and 49% of men, with some significant sex differences (Table 6); 12% of women and 7% of men discontinued MRD therapy for side effects (*p* = 0.017). Mouth or temporomandibular joint pain was responsible for 60% of treatment discontinuations (no difference between men and women).

**Table 6:** Side effects

% Patients	Women	Men	*p* value
Events			
Tooth pain	18	15	NS
Temporomandibular joint pain	25	20	NS
Occlusion change	19	15	NS
Gum irritation	21	10	0.016
Hypersalivation or mouth dryness	6	9	NS
Dental mobility or migration	12	5	0.045
MRD discontinuation	12	7	0.017
Severe side effects	15	13	NS

*MRD* mandibular repositioning device, *NS* not statistically significant

## Discussion

To the best of our knowledge, few studies have compared MRD treatment efficacy in women and men. All patients showed good compliance with therapy, but the treatment success rate was higher in women than in men, particularly in severe OSA. Complete response was also more common in women vs. men, across a range of AHI thresholds.

Previous data on sex differences in MRD effectiveness have reported conflicting results. One prospective observational study suggested that MRD effectiveness was greater in women vs. men [14], while data from a large retrospective cohort of OSA patients treated with an MRD did not find any link between sex and MRD treatment outcome [15]. Better MRD efficacy in women in our OSA cohort could be related to anthropometric and OSA characteristics. At baseline, BMI, waist, and neck circumferences were significantly lower in women, and during MRD use there was a tendency for reduced BMI only in women and increased waist circumference only in men. Obesity is known to be different between genders, with more central obesity in men. In a sleep clinic sample of women with OSA, fat in the neck region had a direct influence on airway patency, explaining 33% of the between-sex variance in AHI [16]. In addition, higher BMI has been associated with lower MRD efficacy in some studies [17], particularly in men [14], and neck circumference may also predict MRD effectiveness [18]. This was confirmed in our study, where multiple regression analysis identified smaller neck circumference at baseline as a statistically significant independent predictor of MRD success in women. Men have a longer, softer oropharynx and a larger, fatter, more posterior tongue, increasing the probability of upper airway collapse [19, 20]. The upper airway in men was found to be more collapsible than that in equally overweight/obese women [10]. These factors, not specifically investigated in our analysis, support a role for anthropomorphic and physiological factors in sex differences in the response to MRD treatment of OSA.

Several studies have reported a lower success rate with MRD therapy in patients with severe OSA [17], but others have found similar response rates across all OSA severities [11, 21]. In our clinical sample, baseline OSA severity was similar in men and women and the proportion of patients with severe OSA was similar in the two groups, but treatment response in the severe OSA group was significantly better in women vs. men. Baseline AHI and HI were similar in men and women in our study, but AI was significantly lower in women. During MRD treatment, AI decreased significantly in men and women, but women continued to have a significantly lower AI. In patients with severe OSA, HI decreased to a significantly greater extent in women vs. men. The lower AI at baseline in women is consistent with data showing that middle-aged women have fewer apneic events compared with men of same age and BMI [22, 23]. A lower AI and larger decrease in HI contributed to the greater reduction in total respiratory events in women during MRD therapy. We have previously shown that both AI and HI were significant predictors of MRD treatment success in this population [11]. However, when analyzing men and women separately, decreases in AI and AHI were only independent predictors of treatment success and complete response in men. This could indicate that, in contrast to men, MRD efficacy in women is independent of OSA severity at baseline.

In this study, positional OSA did not appear to be associated with MRD success in women because supine and non-supine AHI were similar at baseline and decreased significantly with no significant differences between women and men. Effects of MRD treatment on positional OSA are controversial. Several studies have suggested that supine OSA was a predictor of MRD efficacy [24, 25]. Others have reported that supine-dependent OSA predicted MRD treatment success after controlling for other factors, whatever the gender [26] or only in men [14], suggesting that positional OSA is less of an issue for women. In contrast, MRD efficacy was not affected by supine-dependent OSA in some studies [17, 27]. These conflicting results may be explained by the ability of different MRD devices to stabilize the lower jaw in a forward position during supine position sleep [28]. Most, but not all, position-dependent OSA patients appear to maintain positional dependency during MRD therapy, so MRDs might provide additional therapeutic effect in terms of sleep position [29]. This was the case in our population, with time spent in the dorsal position decreasing significantly during MRD treatment in women with severe OSA, and time in the non-dorsal position was significantly longer in women. In contrast, time in the dorsal position increased significantly in men. Less time spent in the dorsal position in women may also contribute to the greater AHI reduction and greater decrease in arousals for severe OSA women during MRD therapy in our analysis. It is well known that the dorsal position promotes respiratory events and lateral position is considered to be protective against apnea [30].

OSA has a well-known deleterious impact on quality of life and functional status. When OSA severity and obesity are similar, women report lower health status than men [31]. In this study, quality of life improved significantly, and to a similar extent, in men and women. Improvements in quality of life in women during MRD therapy are similar to those reported with CPAP [32]. Daytime sleepiness and fatigue were also similarly improved in men and women during MRD therapy, irrespective of OSA severity, and sleep structure was maintained.

Baseline OSA symptom severity was similar in men and women, as has been reported previously [33]. In contrast, sex differences in OSA symptoms and severity have also been documented. Women report typical OSA symptoms (e.g., snoring) less often than men and are more likely to report general symptoms (e.g., fatigue, headache) [19, 34]. In our study, MRD therapy significantly reduced key OSA symptoms in men and women, and reduced morning headache to a greater extent in women than in men. Although women had a higher rate of hypothyroidism at baseline, all were receiving treatment for this condition and the rate was similar to that reported in the general population [34].

Mandibular advancement is associated with enlargement of the velo-pharynx and increased mandibular protrusion produces greater reductions in the AHI. We found no differences between men and women in the number of titration visits, degree of protrusion, and final mandibular advancement, but ability to protrude the mandible was an independent predictor of MRD treatment success in women only. However, it is interesting to note that a high percentage of women (42.9%) vs. men (21.2%) had type 2 angle malocclusion, irrespective of OSA severity. Greater overbite has been described as a significant predictor of treatment success, predisposing patients with class II division 2 malocclusions to a higher success rate [35]. This could have contributed to the higher treatment success rate in women in our study. Women with no previous CPAP therapy, experiencing MRD as first treatment, had higher therapy success rates, suggesting that MRD may be the best treatment for women, even in severe OSA.

Side effects were relatively common, but most were not severe. In our study, women who experienced side effects were more likely to discontinue therapy than men. Careful dental examination before MRD therapy is recommended, especially in women, to improve tolerability and maximize treatment adherence.

This study had some limitations. The design was observational, and there was an imbalance in the number of men and women, consistent with existing OSA prevalence data. Menopausal status of women was not documented, but the number of women aged > 60 years (when most women are post-menopausal) was low. PSG was performed in only 149 subjects and OSA severity by sleep stage was not documented so we could not determine whether these differed between women and men, as described previously [9]. Study strengths include the lack of existing data in this area, and the inclusion and follow-up of patients by a multidisciplinary sleep and dental team. In addition, only CAD/CAM MRD devices were used and these were custom-made for each patient.

In conclusion, our results show that CAD/CAM MRD is an effective treatment option in women with OSA of any severity, particularly those with severe OSA, due to some specific OSA phenotypes in women. Predictors of treatment success varied between men and women. These gender-specific differences in the response to MRD treatment need to be taken into account when deciding on the most appropriate therapeutic strategy for an individual OSA patient.
